# First- and second-line treatment of advanced metastatic non-small-cell lung cancer: a global view

**DOI:** 10.1186/1753-6561-2-s2-s3

**Published:** 2008-09-24

**Authors:** Nicholas Thatcher

**Affiliations:** 1 Division of Cancer Studies, Faculty of Medical and Human Sciences, University of Manchester, Department of Medical Oncology, Christie Hospital NHS Trust, Wilmslow Road, Manchester, M20 4BX, UK

## Abstract

Treatment of non-small-cell lung cancer is dependent on disease stage. For patients with metastasis or locally advanced disease, the importance of finding therapeutic schemes that may benefit this population is important. This review discusses therapeutic options for first- and second-line treatment in patients with advanced non-small-cell lung cancer. According to current data, the combination of two cytotoxic agents is the optimum first-line treatment for patients with non-small-cell lung cancer and performance status of 0–1. Addition of bevacizumab has shown to provide an even longer survival and to increase response rate. Within the first-line setting, erlotinib appears to be effective in the treatment of elderly patients who would not derive a benefit from standard chemotherapy or those refusing standard chemotherapy. The administration of erlotinib as first-line maintenance therapy is being assessed. There are currently three drugs approved for second-line treatment of patients with advanced non-small-cell lung cancer after failure of first-line chemotherapy. These drugs have proven to be effective in phase III trials. In the phase III trial BR.21 study, the response rate was 8.9% in the erlonitib group, and less than 1% in placebo; median response duration was 7.9 months and 3.7 months, respectively; and the median survival was 6.7 months and 4.7 with erlotinib and placebo, respectively. One-year survival was 31% and 21% with erlotinib and placebo, respectively. In addition, the BR.21 trial revealed that significantly greater improvements in overall quality of life and in both physical and emotional functioning were observed in the erlotinib arm as compared with the placebo arm. Erlotinib is not significantly associated with hematologic adverse effects. Erlotinib is administered orally, and does not require concomitant administration of other drugs, thus causing patients less inconvenience. Analysis of data from different subgroups included in the BR.21 trial show that overall survival is similar among women and men, among patients with adenocarcinoma and epidermoid carcinoma or Asian patients compared with other ethnicities. Combination of erlotinib and bevacizumab in the second-line treatment of patients with advanced disease has been evaluated as anti-angiogenic properties. This combination therapy has provided promising results which should be confirmed in future studies.

## Background

Non-small-cell lung cancer (NSCLC) accounts for approximately 85% of all cases of lung cancer, the most common cause of death from cancer in men and second only to breast cancer in women[1,2]. Treatment is dependent on disease stage [1]. Surgery is the treatment of choice for early-stage localized disease. Multimodal therapy remains the norm for patients with locally advanced disease, and patients with advanced metastatic disease derive a benefit from palliative chemotherapy. About 40% of patients with NSCLC present at an advanced stage, with metastasis or locally advanced disease, which underscores the importance of finding therapeutic schemes that may benefit this large patient population. When selecting the treatment for these patients it is of utmost importance to take into account their performance status (PS), which should be considered an important determinant of outcome [3].

Therapeutic options for first- and second-line treatment in patients with advanced or metastatic NSCLC are discussed below.

## First-line treatment

Combination chemotherapy, usually platinum-based, is currently the first-line therapy of choice [1,4]. Based on various studies, doublet regimens combining cisplatin or carboplatin with paclitaxel, gemcitabine, docetaxel, vinorelbine or irinotecan are administered. The choice of the combination components varies in different countries. For instance, in Europe cisplatin or carboplatin are administered in comparable proportions in combination with gemcitabine; while in the U.S. a combination of a platinum compound and paclitaxel or docetaxel is more common. Vinorelbine is indicated less often, and irinotecan is mainly used in Japan.

Various studies have shown similar degrees of efficacy among different combinations in the treatment of advanced NSCLC. For instance, in a randomized trial including over 200 patients in each study group, Kelly *et al *compared paclitaxel plus carboplatin with vinorelbine plus cisplatin and found similar efficacy with both combinations [5]. Another study compared 4 different combinations with cisplatin or carboplatin plus paclitaxel (administered with cisplatin as reference combination), docetaxel or gemcitabine in 1207 patients, and none of the four combinations was found to offer a significant advantage over the others in this indication [6]. In Japan, cisplatin plus irinotecan was compared with cisplatin or carboplatin plus paclitaxel, gemcitabine or vinorelbine, and no significant differences were found either among the different therapeutic regimens [7].

### First-line treatment for elderly patients

In various regions of the world, including the United Kingdom and Mexico, elderly patients (i.e., 70 years of age or older) suffering from advanced NSCLC constitute a special therapeutic challenge [8]. This population shows lower tolerance to adverse effects associated with platinum therapeutic regimens, which in many cases may be contraindicated due to impaired organ function and comorbid conditions [9,10].The ELVIS trial, a randomized double-blind study, evaluated the effect of vinorelbine on quality of life and survival of patients aged 70 or older, as compared to best supportive care [11]. According to data from the 161 patients included in the analysis, vinorelbine showed superiority over best supportive care, with a 1-year survival rate of 32% vs. 14% and a potential improvement in quality of life. Later, also in Italy, the MILES study, a multicenter trial, compared single-agent chemotherapy with vinorelbine or gemcitabine versus polychemotherapy with vinorelbine plus gemcitabine. This trial analyzed data from 698 patients aged 70 years or older (median age, 74; patients > 75 years of age, 39%), and found no differences in survival (1-year overall survival rate, 28%–38%) among the 3 therapeutic regimens, although the combination was found to be more toxic than single agents [12]. Based on results from these and other studies, an expert panel agreed that single-agent chemotherapy with vinorelbine, gemcitabine or a taxane is the optimal treatment for this population [8]. In addition, there is evidence that combinations including certain compounds may be of benefit and well tolerated in elderly patients and few comorbid conditions [12,13]. However, since these are not the most common health conditions in elderly patients, the most frequent indication is to administer the above mentioned single-agent therapies [13].

Another first-line single-agent therapeutic option of benefit to elderly patients with advanced NSCLC is erlotinib administration. Results from a phase II multicenter open-label trial including 80 patients aged 70 years or older with NSCLC and no previous chemotherapy were recently published [14]. Median survival was 11 months, and 1-year survival was 46%, although an objective response was found in 10% of patients. However, 41% of patients remained with stable disease for at least 2 months.

As regards erlotinib, when considering the use of this type of therapy, emphasis should not be placed on objective response rate (since it is usually low) but on survival. It should be noted that in this open-label study median survival and 1-year survival rates are similar, and even higher, than those reported with chemotherapy [8].

Other data related to erlotinib administration as first-line therapy for advanced NSCLC include those presented by Sandler et al. [15] in a study of 127 patients with bronchioloalveolar carcinoma, with a 29% objective response rate. In another phase II trial including 53 unselected patients, objective response rate was 23% (similar to that obtained with chemotherapy), median survival was 60 weeks, and median time to disease progression was 84 days [16]. A randomized trial is required to confirm results of this study. Finally, a phase II trial included 72 chemotherapy-naïve patients (median age 74.4 years) with advanced NSCLC and PS 2 [17]. In these patients, who have a worse prognosis than those with PS 0–1, objective response rate was 8%, median survival was 5 months, and 1-year survival was 22%.

### Erlotinib as first-line maintenance therapy

Another study assessing erlotinib in the first-line treatment of NSCLC is the Sequential Tarceva in Unresectable Non-Small-Cell Lung Cancer (SATURN) trial, recently closed to enrolment of patients. This phase II trial evaluates the efficacy of erlotinib as maintenance therapy for patients with advanced NSCLC and no disease progression following four cycles of chemotherapy. After initial chemotherapy, patients are randomly assigned to erlotinib or placebo; the primary endpoint is progression-free survival. Preliminary results are expected to be reported by the end of 2008 [1].

### Other therapeutic options

Another promising drug for first-line treatment of advanced NSCLC is bevacizumab, a monoclonal antibody directed against the vascular endothelial growth factor, which when administered concomitantly with chemotherapy (carboplatin plus paclitaxel), and as maintenance single-agent therapy following six cycles of combination chemotherapy, increased overall survival from 10.3 months to 12.3 months and response rate from 15% to 35% [1]. Progression free survival was increased after initial chemotherapy with cisplatin and gemcitabine.

## Second-line treatment

There are currently three drugs which have been approved in the U.S. and Europe for second-line treatment of patients with advanced NSCLC after failure of first-line chemotherapy: docetaxel, pemetrexed and erlotinib. These drugs have proven to be effective in phase III trials [18]. A phase III randomized trial including 571 patients with advanced NSCLC previously treated with chemotherapy showed that pemetrexed was comparable in terms of clinical efficacy to docetaxel; in both arms, 1-year survival rates were 29.7% [19]. Median progression-free survival was also comparable in both arms (2.9 months), and median survival time was 8.3 versus 7.9 months for pemetrexed and docetaxel, respectively (non significant difference).

The BR.21 study was a phase III randomized double-blind placebo-controlled trial assessing the efficacy of erlotinib treatment of patients with advanced and chemotherapy-refractory NSCLC [20]. A total of 731 patients were randomly assigned in a 2:1 ratio to receive erlotinib or placebo. Patients were required to have received one or more previous lines of chemotherapy; in addition, they could have a PS score of 0–3. At the time of the study, pemetrexed was not available, and there was concern about the toxicity and effectiveness of further chemotherapy after failure of standard chemotherapy in some patients, therefore it was reasonable to compare erlotinib with placebo [21,22]. The primary endpoint of the study was overall survival; secondary endpoints were response rates, stable disease rate, duration of response, time to disease progression and quality of life. Forty-nine percent of patients had received two previous chemotherapy regimens, and 93% had been treated with platinum-based regimens. Response rate was 8.9% in the erlonitib group, and less than 1% in the placebo group; median response duration was 7.9 months and 3.7 months, respectively. Median survival was 6.7 months with erlotinib and 4.7 months with placebo (p < 0.001). One-year survival was 31% with erlotinib and 21% with placebo. Although the 10% observed difference in 1-year survival appears to be small, it should be taken into account that initial studies on platinum compounds in the first-line treatment setting also showed a 10% difference in 1-year survival as compared with supportive treatment. Results presented in ASCO 2007 (Annual Meeting of the American Society of Clinical Oncology), obtained from the TRUST (TaRceva lUng cancer Survival Treatment) trial, which includes patients cared for in real clinical practice, are very similar to those reported in the BR.21 trial, and survival curves follow the same pattern [23]. This underscores the fact that in everyday practice erlotinib offers the same advantages as those observed in clinical trials.

### Quality of life

When treating patients with cancer, physicians seek the best therapeutic results. However, patients do not always have exactly the same priorities when setting therapeutic goals. Generally, patients wish to experience symptom improvement, few toxic effects and better quality of life. They also expect to live longer.

A study conducted by Silvestri et al., which enrolled 81 patients with advanced NSCLC already treated with chemotherapy, showed that many patients would choose chemotherapy treatment for its effects on quality of life rather than for the potential survival offered by the treatment [24]. Only 25% would choose chemotherapy if benefits were exclusively related to survival. More recently, it was noted that nearly 75% of patients would choose the therapeutic regimen according to related adverse effects [25]. Surprisingly, the adverse effect most taken into account when making the decision was chemotherapy-related nausea. Similar results were observed in other regions of the world such as Japan [26].

As previously mentioned, the BR.21 trial evaluated the effect of erlotinib treatment on quality of life [20,27]. The European Organization for Research and Treatment of Cancer (EORTC) validated questionnaires were used, and significantly greater improvements in overall quality of life and in both physical and emotional functioning were observed in the erlotinib arm as compared with the placebo arm. In addition, a significantly longer median time to deterioration for cough, dyspnea and pain was recorded. These data show that erlotinib not only prolongs survival in these patients but also improves their symptoms and quality of life.

When effects of the other therapeutic options available for second-line treatment are evaluated, treatment with docetaxel is found to have shown some improvement in pain and fatigue, but not in overall quality of life [28]. In addition, these effects were achieved among patients receiving the highest dose of docetaxel, which later had to be discontinued due to dose-related severe adverse effects, including mortality.

In the study comparing pemetrexed and docetaxel, no significant differences among both drugs were found in overall burden of symptoms or in quality of life, despite the more favorable profile of adverse effects of pemetrexed [19].

### Adverse effects

Erlotinib is not significantly associated with hematologic adverse effects [20], while 40% of patients treated with docetaxel and 5.3% of those receiving pemetrexed experience severe neutropenia, with 12.7% and 2% having febrile neutropenia, respectively [19]. These effects significantly affect not only potential survival but also quality of life.

Most commonly reported erlotinib-related adverse effects were rash and diarrhea [20]. Diarrhea is not a major problem, since it may be managed with standard therapies. Rash was found in around 1 out of 20 patients receiving erlotinib. When rash develops on the face, it may cause complications, but it is usually transient and does not prevent continuation of treatment. In some cases it may be necessary to reduce the dose of the drug, but ideally it should not be discontinued. Moreover, there are data suggesting that patients experiencing rash tend to have longer survival [29].

Another aspect that should be considered is the need of premedication and invasive procedures for the administration of the various available therapies [30]. Both docetaxel and pemetrexed require administration of premedication or other related drugs, and are administered intravenously; whereas erlotinib is administered orally, and does not require concomitant administration of other drugs, thus causing patients less inconvenience.

### Relationship between objective response and benefits

In the analysis of data from the BR.21 trial related to patients not showing objective response, i.e., those showing stable disease or disease progression, it is found that even without evidence of objective response, there is an improvement in survival (median survival 8.25 months, as compared to 6.8 months with placebo; hazard ratio (HR) 0.82; CI 0.68–0.99; p = 0.037).

Apparently, erlotinib reduces the pace of disease progression, and this is associated with longer survival.

### Efficacy according to patients' specific characteristics

When comparing different subgroups of patients treated with the different therapeutic options now available, we see that in the BR.21 trial, despite having enrolled more patients with a PS score of 2 and patients with two or more previous chemotherapy cycles, erlotinib has similar effects on survival to those reported with docetaxel and pemetrexed. Patients with ECOG score 0–1 and one prior regimen show a survival of 9.42, 9.15 and 9.45 months with erlotinib, docetaxel and pemetrexed, respectively [19,20,30].

Moreover, the beneficial effect of erlotinib is observed in all subgroups of patients examined separately, such as women or men, smokers or non-smokers, and with different histopathological features, as shown by the analysis using Forest plots. However, there is less benefit among smokers, and this could be related to the fact that these patients have lower peak plasma concentration of the drug than non-smokers. In addition, it should be noted that rash is also correlated with maximum peak blood levels of erlotinib.

Other characteristics, such as previous chemotherapy and therapeutic response, time from diagnosis, EGFR status, and ethnicity, do not cause survival benefit to erlotinib.

There is some tendency to consider that erlotinib is only effective in certain subgroups of patients, such as women or Asians, or among patients with adenocarcinoma. Analysis of data from subgroups included in the BR.21 trial show that overall survival is similar among women and men (HR 0.8 for both populations) [20]. Likewise, survival is similar among patients with adenocarcinoma and epidermoid carcinoma (HR 0.7 and 0.67, respectively). When comparing Asian patients with other ethnicities, results are similar (HR 0.6 and 0.8, respectively). It should be noted that these data were obtained from a relatively small number of patients.

A most important group is that of male smokers with epidermoid carcinoma. In this population, HR in the erlotinib group (n = 100) is 0.66, and median survival was 5.5 months, as compared to 3.4 months in the placebo group (n = 57) [31].

Another significant aspect is that of EGFR mutations. It has been argued that only patients with certain specific mutations respond to erlotinib. However, survival of patients with such mutations (exon 19 and exon 21) who received placebo during the BR.21 trial is found to be 9.1 months, as compared to 3.5 months in the remaining patients with wild-type EGFR assigned to the placebo group. Thus, these mutations should be considered to be a prognostic factor, although they can be associated with better objective tumour response to the agent, survival is increased in both mutation and wild type patients [32].

As regards rash, in the TRUST trial 11% of patients required a dose reduction due to this adverse effect [23]. This figure is not markedly different from that observed in the BR.21 trial (6%). There are no significant differences either between both trials as to the proportion of patients developing any kind of rash.

#### Future options

Combination of erlotinib and bevacizumab in the second-line treatment of patients with advanced NSCLC has been evaluated, since the former, apart from its effect on EGFR, has antiangiogenic properties. The evaluation of this combination yields promising results [33]. In another phase II trial including patients previously treated with chemotherapy who were assigned to erlotinib plus bevacizumab, bevacizumab plus chemotherapy, or chemotherapy alone, a 1-year survival rate of 57.4% was observed with the first combination [34].

Phase III studies are required to further assess these promising data.

## Conclusion

Erlotinib is an adequate therapeutic option for the second-line treatment of patients with advanced NSCLC.

This drug has shown to have similar efficacy to chemotherapy, with better tolerability and proven positive effects in terms of survival, quality of life and symptom improvement.

Since there are no significant differences in survival among the subgroups, selection of patients who should receive erlotinib is not justified.

Erlotinib administration should continue to be evaluated as a first-line treatment in elderly patients with advanced NSCLC, in those who do not derive a benefit from standard chemotherapy due to comorbid conditions, or in those patients refusing conventional chemotherapy.

Erlotinib administration as first-line maintenance therapy is being evaluated.

Combination of erlotinib and other agents, such as bevacizumab, has provided promising results that should be confirmed in future studies.

## Competing interests

Dr. Nicholas Thatcher has received honoraria for advisory boards, lectures and research support from AstraZeneca, Lilly, Sanofi-Aventis, Roche.
